# Effects of a Virtual Reality Game on Children’s Fear and Anxiety During Dental Procedures (VR-TOOTH): A Pilot Randomized Controlled Trial

**DOI:** 10.1089/jmxr.2024.0021

**Published:** 2024-07-09

**Authors:** Wenjia Wu, Marie-Eve Asselin, Nicole Hung, Pascale Ouimet, Olivier Fortin, Christine Genest, Maxime Francoeur, Estelle Guingo, Kate St-Arneault, Annie Sylfra, An Kateri Vu, Janick Carmel, Laurence Lessard, Stephany Cara-Slavich, Kathryn DeKoven, Julie Paquette, Hunter Hoffman, Sylvie Le May

**Affiliations:** 1 Department of Dental Medicine, Centre Hospitalier Universitaire Sainte-Justine, Montreal, Canada.; 2 Faculty of Dental Medicine, University of Montreal, Montreal, Canada.; 3 Azrieli Research Center, Centre Hospitalier Universitaire Sainte-Justine, Montreal, Canada.; 4 Faculty of Nursing, University of Montreal, Montreal, Canada.; 5 Trauma Studies Centre, Le Centre intégré universitaire de santé et de services sociaux de l’Est-de-l’Île de Montréal, Montreal, Canada.; 6 UER en sciences de la santé, Université du Québec en Abitibi-Témiscamingue, Quebec, Canada.; 7 Department of Anesthesia, Centre Hospitalier Universitaire Sainte-Justine, Montreal, Canada.; 8 Virtual Reality Research Center, University of Washington, Seattle, Washington, USA.

**Keywords:** virtual reality, pediatrics, children, dentistry, procedures, fear, anxiety, dental fear and anxiety, children with special needs, salivary alpha-amylase

## Abstract

Dental fear and anxiety (DFA) is a condition affecting approximately a quarter of children and adolescents and can cause a lack of cooperation during dental visits. Virtual reality (VR) use during dental care is a potential nonpharmacologic adjunct to better manage DFA in children with special health care needs (SHCN) undergoing dental procedures. This study aims to assess the feasibility and acceptability of VR in pediatric patients with SHCN undergoing dental procedures, as well as their parents and health care providers (HCPs), and evaluate the effect of VR on children’s DFA during appointments. This pilot randomized controlled trial conducted at CHU-Ste Justine in Montreal followed a parallel design where participants were randomized into two groups: control (wall-mounted TV) and experimental (VR). The primary outcomes were recruitment rates and completion rates of procedures. DFA was assessed using the Venham Anxiety and Behavior Rating Scales (VABRS) and physiological biomarkers. Descriptive and nonparametric mean comparison tests were used for analyses of demographics, clinical variables, satisfaction, and VABRS. Of the 36 patients approached for recruitment, 25 (69.4%) accepted to participate (13 randomized to the VR group and 12 to the control group). The mean age of participants was 10.2 (±2.8) years and 64% were males. Overall, 77% (10/13) of participants in the VR group tolerated the headset during the procedure. Parental and HCP satisfaction was high: all HCPs indicated they would use VR again; all parents rated the VR intervention with a score of 8/10 or higher. There was no significant difference between groups on VABRS and physiological biomarkers (*p* > 0.05). This pilot study showed that VR was a feasible and acceptable tool for SHCN children during dental treatments. Parents and HCPs were highly satisfied. However, future studies are needed to verify the impact of VR on children’s fear and anxiety during dental procedures.

## Introduction

Dental fear and anxiety (DFA) is a condition that affects approximately 13.3%–29.3% of children and adolescents and is a significant cause of patients avoiding dental care, leading to a lower oral health-related quality of life.^1–3^ Although the etiology may be multifactorial, a previous traumatic dental experience is the most predictive factor for DFA.^4,5^ Short-term distress during appointments that is not managed properly can accumulate into poor dental experiences, and in turn, reinforce DFA into adulthood.^6–8^

Dental patients with special health care needs (SHCN) are defined as patients requiring additional time and special consideration when receiving treatments because of medical, physical, cognitive, or developmental conditions.^9^ Children with SHCN face more barriers to dental care than the overall population and experience more DFA, which can result in more difficult dental visits.^9^,^10^ The importance in providing well-rounded care and making each dental visit a positive one for pediatric patients with SHCN is crucial in promoting a good oral health routine, as well as improving their oral health when transitioning into adulthood.^9^

Factors in the dental setting that trigger DFA include the loud sounds of dental instruments, the presence of strangers examining the oral cavity, injections, and the fear of pain.^11^ Pharmacological agents, combined with light-to-moderate sedation, or general anesthesia can also be considered for noncooperative patients, which are often time consuming and at a higher cost and health risk.^1^ In the current literature, audiovisual distractions such as tablets/TV screens have been used as additional techniques to traditional tell-show-do distraction, with overall positive results.^12^ However, there is a lack of interactivity of these techniques, and as a result, produces less immersive environments of distraction for children.^13^ Lack of patient cooperation because of DFA often obliges dentists treating pediatric populations to end appointments prematurely, and sometimes without completion of the planned procedure. Particularly when treating children with SHCN, some patients have hypersensitivity to external stimuli, such as loud noises, aversion to specific tastes, and difficulty straying from usual daily routines.^14,15^

Virtual reality (VR) is defined as an artificial environment that is experienced through sensory stimuli.^16^ Commonly used in the medical field to help distract patients during unpleasant procedures such as vaccination, cast removal, and short bedside interventions, it has proven to be effective at decreasing procedural anxiety and providing a more positive experience for patients.^17^ Among the limited existing literature, the use of VR to manage DFA during dental procedures have shown positive results. A recent clinical trial by Alshatrat et al.^13^ concluded that VR is an effective tool in reducing anxiety in young children during dental procedures. Moreover, a study by Du et al.^18^ showed that VR was preferred to traditional behavior guidance for the management of DFA in children during dental extractions. However, clinical VR research in pediatric dentistry is very limited, especially in pediatric patients with SHCN. A study by Pagano et al.^14^ showed that the use of augmented reality was well suited for patients with autism spectrum disorder (ASD) in preparation for their dental visits.

A clinical study on the use of VR during dental appointments in pediatric patients with SHCN would allow a better understanding of the feasibility and effects of VR on DFA in this population and possibly facilitate dental procedures.

### Aims of the study

The aims of this pilot randomized controlled trial were 2-fold: (1) To assess the feasibility and acceptability of VR immersion as a tool to reduce DFA in pediatric patients with SHCN undergoing dental procedures and (2) to gain insight on parents’ and health care providers’ satisfaction with the use of VR during dental appointments.

### Objectives

The primary research objectives were to determine the following: (1) The feasibility and acceptability of VR distraction for children with SHCN requiring dental procedures. (2) To evaluate parents’ and health care professionals' satisfaction between both groups (*VR group vs. Cartoon on a muted wall-mounted TV group*). (3) To observe the preliminary effects of VR distraction on DFA.

The secondary objectives of this study were to compare between both groups:

1. Physiological parameters (heart rate and oxygen saturation);
2. Occurrence of side effects;
3. Length of dental procedure;
4. Number of retakes of dental procedures; and
5. Mean difference in the level of salivary alpha-amylase.

## Methods

### Design

This pilot randomized controlled trial study followed a parallel design, including two groups: A control group (Cartoons on a muted wall-mounted TV) and an experimental group (VR game through a headset). Approval by the Ste-Justine Hospital research board of ethics No. 2023-4985 was obtained in March 2023. The trial was registered on clinicaltrials.gov (NCT05898100).

### Sample and setting

This pilot study included 25 participants, allocated and randomized in an equal ratio per group. Recruitment was carried out at the dental clinic of the Centre Hospitalier Universitaire Sainte-Justine, a pediatric university teaching hospital in Montreal, Canada. This clinic mainly serves patients with SHCN such as craniofacial abnormalities, ASD, children battling cancer, and others. Pediatric patients with SHCN represent about 80% of the total clientele while the rest is comprised of otherwise healthy patients but presenting with dental traumas and other emergencies.

### Inclusion criteria

Children and their parents were invited to participate in the study if they met the following criteria: (1) Aged 6–17 years; (2) received the dentist’s recommendation to participate. Children who are uncooperative (e.g., needing active or passive restraints) were not given recommendation by the dentist to participate; (3) required to undergo any dental procedure; and (4) accompanied by a parent or a legal guardian who can understand, read, and write in either French or English. Considering this was a pilot study, any dental procedure was eligible, allowing us to investigate the feasibility of VR during various dental procedures.

### Exclusion criteria

Participants were excluded from this study if they met the following criterion: (1) suffer from epilepsy or any other conditions preventing them from using VR (e.g., recent eye surgery). Patients with a strong history of motion sickness were not excluded from the study, but this information was documented. Since this was a pilot study, we used a convenience sample, thus patients with non-SHCN were not excluded from the study, but this information was documented. In fact, only one participant was non-SCHN. The use of medication (opioid and nonopioid analgesic, antiemetic, anxiolytic, or any other drugs) before the procedure within the last 4 hours did not exclude participants from this study, but this information was collected in the preintervention questionnaire. Patients who previously had dental treatments at the same clinic were not excluded.

### Randomization and allocation

Randomization was performed through the Research Electronic Data Capture (REDCap) system. Allocation to either intervention was randomized by an independent biostatistician from the Applied Clinical Research Unit (URCA). To equalize participants in both arms, permuted block randomization with a randomly selected block sizes design was used to randomize participants for their intervention.^19^ Access to the randomization list was only granted to the biostatistician and allocation was concealed using REDCap to control selection bias.

## Interventions

### Control treatment

The control group received a care-as-usual approach. This included viewing a cartoon on a muted wall-mounted television and the use of pharmaceutical treatment during the procedure, such as the use of injected local anesthesia if needed. In the event of noncooperation during the appointment, any re-take or re-scheduling of appointments were documented. Only one parent could be present in the room during the procedure, as part of the clinic’s usual protocol and their presence was documented. Children allocated to the control group were offered the possibility to try the VR game after the study period.

### Experimental treatment

**FIG. 1.**
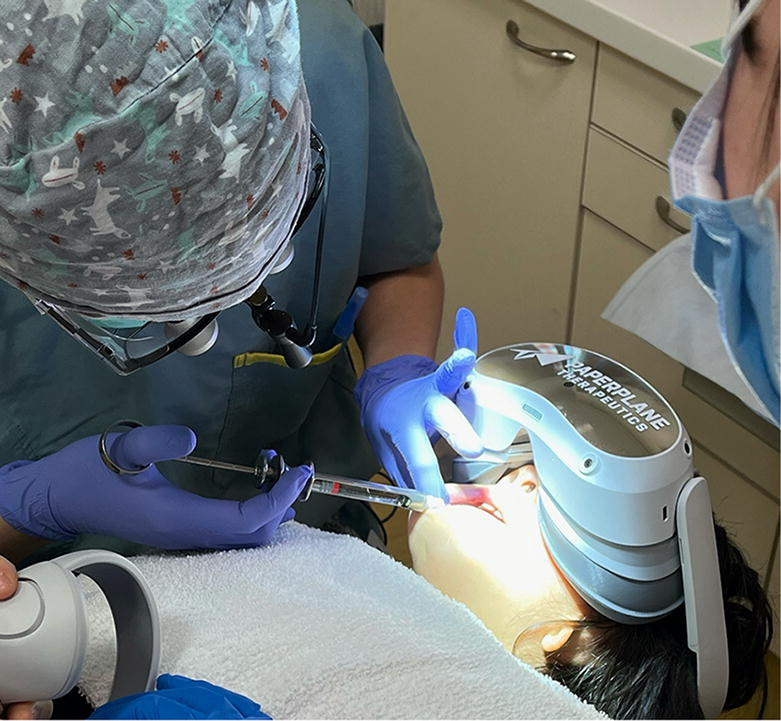
Virtual reality (VR) headset in use during the dental procedure.

The experimental group received the VR video game Dream Dental developed by Paperplane Therapeutics^®^. It is an easy-to-play, no-success immersive VR video game, where children use a remote in one hand to throw balls at targets such as balloons, trolls, and diamonds to gain points. Dream Dental was designed specifically to be used in a horizontal position that is essential for dental procedures and supported by the Pico Neo 4 VR headset. Dream Dental game uses eye-tracking technology to help the child navigate the same way as head movement normally would in classical VR, making it easier for dental procedures where head movement is restricted. These features also aim to reduce cybersickness. Pharmaceutical treatment during the procedure, such as the use of injected local anesthesia, was used if needed. Figure 1 shows an image of the VR device in use during a dental restorative appointment.

The preliminary version of the VR headset was tested in the clinic on staff before the start of the study—the size and volume do not hinder the delivery of dental care. Children were able to play for the entire duration of the dental procedure. The VR headset simultaneously obstructs the partial view they would normally have of the dental procedure.

### Study time-points

There were three study time points: T0 was at baseline, before the beginning of the dental procedure, T1 was 10 min after the start of the dental procedure, and T2 was at the end of the procedure.

## Measures and Outcomes

### Sociodemographic characteristics

Sociodemographic characteristics were collected before the intervention in the waiting room by the parent or legal guardian present and included information, such as age, sex, ethnicity, and the projected procedure. Other information collected included any medication taken within the last 24 h (name, class, and posology) that could have an impact on the conclusions of the study.

### Primary outcomes

To measure feasibility and patient acceptability, we recorded patient recruitment rates and completion rates of planned procedures.

### Measures of primary outcomes

Parent’s or legal guardian’s satisfaction with the intervention and side effects were assessed using the Visual Analog Scale (VAS), (0–10, where 0 means very dissatisfied and 10 means very satisfied) and following the recommended question by Pediatric Initiatives on Methods, Measurement and Pain Assessment in Clinical Trials (PedIMMPACT): “Considering anxiety relief, side effects, and emotional recovery, how satisfied were you with the intervention used to manage DFA experienced by your child?”^20^

HCPs satisfaction and evaluation of each child’s side effects were self-assessed using a six-question satisfaction questionnaire with a Likert response scale with four options. Participant’s DFA scores were evaluated using the Venham Anxiety and Behavioral Rating Scales (VABRS), a reliable, valid, observation-based assessment by proxy for DFA. It is among the most frequent behavior-scoring instruments for DFA.^21^ The scale has been used as an anxiety rating scale in other studies, which evaluated the efficacy of VR distraction in the management of DFA.^22–24^ Both anxiety and behavior subscales of the VABRS consist of a six-point scale, with six defined behavioral levels that range from 0 to 5. The highest score represents a high level of anxiety or lack of cooperation.^21^ A high degree of reliability was observed for both subscales, even by untrained observers.^21,25^ Measures on the VABRS were obtained at T0, T1, and T2 by the research assistant.

### Secondary outcomes

The secondary outcomes were the following: (1) changes in physiological parameters (heart rate, oxygen saturation, and level of alpha-amylase) during and after the intervention compared with Baseline; (2) occurrence of side effects; (3) procedural length, and (4) rescheduling of procedures in the event cooperation was impossible. We chose to sample salivary alpha-amylase, as a valid stress biomarker.^26–30^ As this was a pilot study, we wanted to evaluate the feasibility of performing this noninvasive sampling procedure within a busy pediatric dental clinic.

### Measures of secondary outcomes

The secondary outcomes were measured as follows:

1. Physiological parameters were continuously measured using a COVIDIEN Nellcor pulse oxygen saturation meter.
2. Occurrences of side effects were collected from arrival on site to discharge from the study using a checklist of common side effects experienced while using VR and related to dental medication.
3. The length of the dental procedure was measured in minutes and documented for every participant.
4. The occurrence of rescheduling of procedures in the event where cooperation was impossible was documented.
5. Measurement of salivary alpha-amylase using a sterile cotton swab was collected at Baseline (T0) and 10 min after the dental procedure (T2).

### Study proceedings including data collection

Participants were identified by the resident dentist through the appointment scheduling system as required by any dental procedure. An individual independent to the study team reviewed the consent form with participants and parents. Written consent by the parents or legal guardian—including the child’s assent—was obtained on arrival at the clinic the day of the procedure. Baseline data collection before the dental procedure took approximately 15 min to complete and included a sociodemographic questionnaire, recording of physiological parameters, and salivary alpha-amylase sampling.

After confirmation of the eligibility to the study, a research assistant logged into REDCap 10 min before the start of the intervention to minimize the risk of bias toward the intervention, obtained the group allocation, and proceeded to inform the participant and parents. Owing to the nature of VR, no blinding was possible to staff, participants, parents, or legal guardians. The VR headset was adjusted to the child’s head size and approximately 5 min was allotted to children to familiarize themselves with the room, equipment, and game before the start of the procedure.

The intervention lasted the entire time of the dental procedure, and the total duration of the procedure was collected for every patient. DFA assessment by proxy, using the Venham Anxiety and Behavioral Rating Scales (VABRS) was performed on site by the research assistant.

As per the clinic’s standard protocol, if a child became restless and cooperation was deemed impossible, he/she was either held by his/her parent for the remaining of the procedure if it could not be safely stopped at that time or if the procedure was considered an emergency. If the procedure could be safely stopped, rescheduling was discussed with parents including the possible need for sedation.

### Statistical analyses

Analyses were conducted using the statistical analysis software SAS (version 9.4; Cary, NC). Descriptive statistics presented by group treatment were conducted for demographic and clinical variables and were used to present sociodemographic and clinical data, parents’ and healthcare professionals' satisfaction levels, and procedural time. We performed descriptive analyses of the sample as well as comparative analyses between the two groups to observe if there was an effect of either intervention, using nonparametric tests. Comparison of dichotomous variables and the occurrence of side effects were assessed using the Cochran–Mantel–Haenszel tests. Analyses were carried out with a significance level (*α*) of 0.05.

## Results

### Participants’ characteristics

**FIG. 2.**
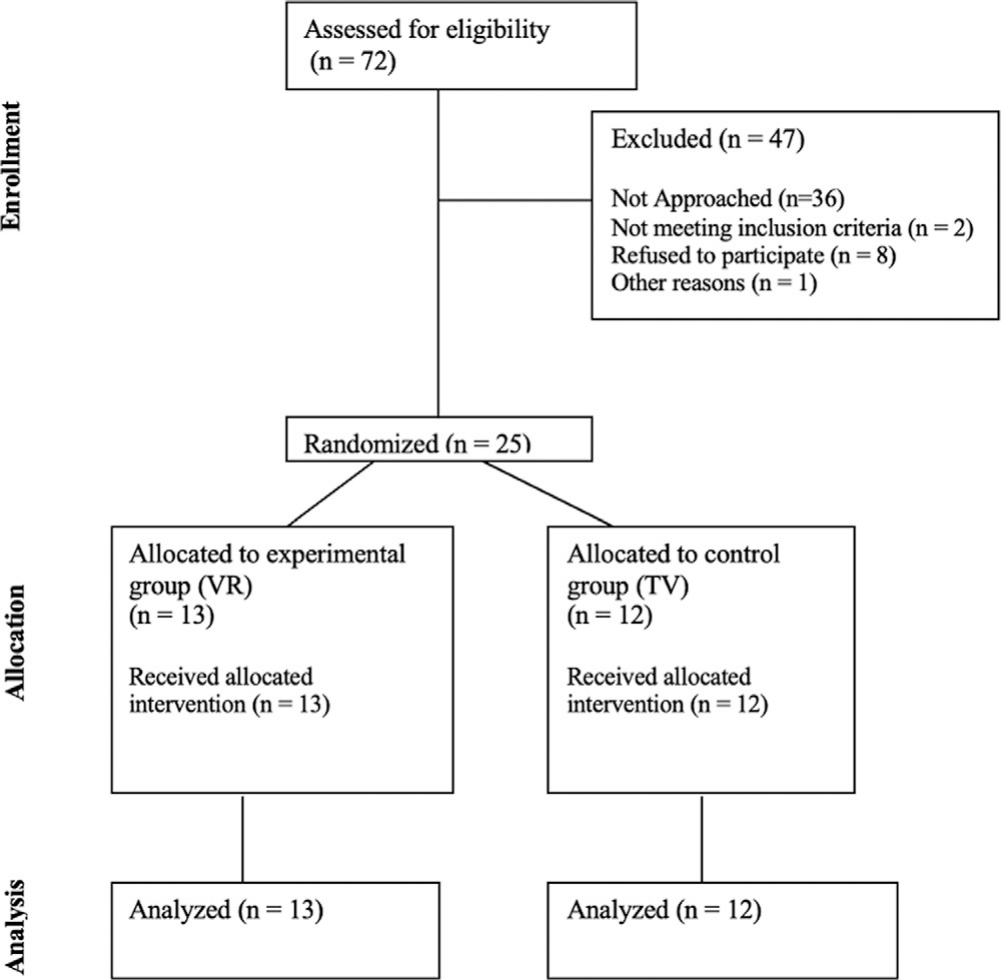
Participants’ flow chart.

A total of 25 participants were recruited (69.4%; 25/36) for this pilot randomized clinical trial during the period spanning from June to August 2023. Figure 2 shows the flow of participants in the study. Participants included more boys (64%) than girls and the mean age was 10.2 ± 2.8 years. No side effects were reported among the participants in either group. The main characteristics of the participants are presented in Table 1.

**Table 1.** Characteristics of the Participants

	Virtual reality (*n* = 13)	Control (*n* = 12)	Total (*n* = 25)
Age (years), mean (±SD)	10.3 (± 3.4)	10.2 (± 2.1)	10.3 (± 2.8)
Sex			
Female	5 (38.5%)	3 (25%)	8 (32%)
Male	8 (61.5%)	8 (66.7%)	16 (64%)
Missing	—	1 (8.3%)	1 (4%)
Ethnic background			
Caucasian	6 (46.2%)	4 (33.3%)	10 (40%)
Asian	1 (7.7%)	—	1 (4%)
Middle Eastern	2 (15.4%)	4 (33.3%)	6 (24%)
Black	2 (15.4%)	1 (8.3%)	3 (12%)
Indigenous	—	1 (8.3%)	1 (4%)
Other	2 (15.4%)	1 (8.3%)	3 (12%)
Missing	—	1 (8.3%)	1 (4%)
Previous dental procedures			
Yes	11 (84.6%)	11 (91.7%)	22
No	1 (7.7%)	—	1
Missing	1 (7.7%)	1 (8.3%)	2
Medical condition			
Behavioral or cognitive (e.g., autism spectrum disorder, attention-deficit/hyperactivity disorder, intellectual disabilities)	3	3	6
Systemic (e.g., childhood cancer, incontinentia pigmenti)	7	5	12
Congenital (e.g., T21, Prader–Willis Syndrome)	1	2	3
Specific conditions (e.g., cleft lip/palate, amelogenesis imperfecta)	5	3	8
No reported medical condition	1	1	2
Missing	1	1	2
Dental procedures^b^			
Complete dental exam	5	7	11
Cleaning	5	5	10
Restoration	4	1	5
Sealant	1	1	2
Extraction	1	2	3
Anxiolytic medication before appointment			
Yes	1 (7.7%)	—	1
No	12 (92.3%)	11 (91.7%)	23
Missing	0	1 (8.3%)	1

a Some participants had multiple medical conditions.

b Some participants received more than one type of treatment.

### Feasibility and acceptability

All 25 recruited participants completed the planned dental procedure. Of the patients, 77% (10/13) tolerated the headset well; however, three participants in the experimental VR group had the headset removed mid-treatment (3/13 participants = 23.1%). Two participants asked for it to be removed, and for one participant the dentist decided to remove it because of impossible cooperation. In all three cases, the treatment was completed without the VR headset. Furthermore, some adolescents expressed that the specific game used was not suitable for their age group; however, they still appreciated it over the clinic’s standard of care intervention: “I find that the VR game wasn't age-appropriate for me, but I still prefer VR over the cartoon on the muted wall-mounted television.” —(translated from French).

### Healthcare professional and parental satisfaction

Parental satisfaction was high in both groups, with a mean rating on VAS of 9.7/10 for both groups. Parents expressed that the introduction of VR in dental clinics was a great idea: “A very enjoyable activity for children, especially those who are afraid of dentists! Good idea!”—(Translated from French).

As for health care professional satisfaction scores, all responded “agreed” or “strongly agreed” that they would use VR again for other dental procedures for kids, that the game was adapted to the dental environment, and that the concept of using VR in pediatric dentistry is worth being studied. Two health care professionals answered that the VR game hindered the delivery of care, while the other 7 “disagreed” or “strongly disagreed” to this statement. Two respondents “disagreed” that VR helped the child cooperate better during the appointment, while the other 7 “agreed” or “strongly agreed.” Of the respondents, 6 HCPs preferred VR immersive distraction while one preferred passive distraction. Overall, the practitioners did not encounter difficulties performing dental procedures with the VR headsets in place, but one professional mentioned that it would be interesting to have a smaller headset for smaller children: “Honestly, it’s a bit bulky, it takes up space and we need to adapt to achieve an ergonomic position… but it’s great for certain patients!”—(Translated from French).

### Preliminary efficacy of VR distraction on dental anxiety and fear

There was no significant difference between groups on anxiety and behavior measured by the VABRS (see Table 2). When analyzed by age groups (6–12 years vs. 13–17 years), for anxiety during treatment *F*-value = 1.295 (Pr[>F] = 0.269) and behavior during treatment *F*-value = 1.144 (Pr[>F] = 0.298), there were no significant differences between groups.

**Table 2.** Mean Anxiety and Behaviour Scores (VABRS) at Each Time-Point

	Baseline (T0)		During the procedure (T1)		After the procedure (T2)	
	VR mean ± SD	Control mean ± SD	VR mean ± SD	Control mean ± SD	VR mean ± SD	Control mean ± SD
Anxiety (0–5)	0.1 (± 0.3)	0.1 (±0.3)	0.8 (±1.7)	0.6 (± 0.9)	0.5 (± 1.5)	0.2 (± 0.4)
*p-*values^*^	*p* = 0.95		*p* = 0.69		*p* = 1.0	
Behavior (0–5)	0.2 (± 0.4)	0 (± 0)	0.8 (± 1.7)	0.5 (± 0.8)	0.4 (± 1.4)	0.1 (± 0.3)
*p*-values^*^	*p* = 0.20		*p* = 0.90		*p* = 1.0	

* Significant at *p* < 0.05.

VR, virtual reality.

### Results of secondary outcomes

Regarding physiological markers of anxiety, there were no significant differences in pulse, oxygen saturation, and salivary alpha-amylase (*p* > 0.05).

Neither the experimental nor control groups reported any side effects during the dental procedures; however, one participant in the control group had missing data to this regard. The difference on the mean procedural time between experimental (16.4 min ± 9.8 min) and control groups (13.8 min ± 4.0 min) was not statistically significant (*p* > 0.05). All participants completed the planned dental procedures, no appointments were rescheduled.

## Discussion

The primary objectives of our study were to evaluate the feasibility and acceptability of VR as an immersion tool for children with SHCN undergoing dental procedures.

Results of this study showed that VR as a distraction method during dental procedures for children with SHCN was feasible and acceptable with a recruitment rate of 69.4% and 77% (10/13) of the patients tolerated the VR headset during all the procedures. Some participants and parents refused participation for several reasons, the main reason being a lack of interest from the child during recruitment. Individual experience with VR varies; some children may love to have an active distraction during dental treatment, while others may prefer to observe the procedure. This reinforces the importance of case selection and tailoring distraction options to the patient. No other issues arose in terms of feasibility.

As for acceptability, all participants completed their planned procedures. However, for 3 (23.1%) participants in the experimental VR group, the VR headset was removed either because of non-cooperation or because the child did not want to continue with the game. With VR masking the surrounding environment, it provides the advantage of avoiding seeing some of the dental instruments, such as the syringe and needle for the administration of the local anesthetic. However, it is more difficult with the VR headset on to explain or show a certain instrument or procedure mid-treatment as clinicians would have performed without the headset in place. This is an aspect to keep in mind for future studies.

Satisfaction from both parents and health care professionals was very high. Since some professionals considered that VR may hinder dental care, a majority thought that it was an intervention worth pursuing research on its impact for children during dental care. VR is a new tool in pediatric dentistry and requires certainly a period of adaptation for both the clientele and dental professionals to consider it as part of their toolkit to distract patients during dental procedures.

Also, our results showed no significant difference between groups regarding the effects on behavior, anxiety, physiological parameters, and alpha-amylase. This differs from recent studies by Felebem et al.,^31^ Shetty et al.,^32^ which both concluded that VR significantly reduced anxiety during dental treatments in children. However, their respective studies had larger sample sizes with healthy, nonmedically compromised children and used self-reported anxiety scales compared to proxy reported as in our study.

Furthermore, patient populations greatly vary between dental clinics, whether it be in a hospital-based setting or in a community setting. The results of the pilot study may not be generalized to all pediatric dental clinics. Moreover, although VR use during dental treatment is a tool that may be useful for certain populations of patients, proper screening and patient selection to use VR is crucial during treatment planning.

### Challenges and limitations

This pilot study was mainly developed to evaluate feasibility and acceptability as the sample size was smaller. However, a larger sample size would provide more power to verify the efficacy of VR for DFA in children with SHCN.

Also, regarding screening, patients were identified by the supervising dentist and considered past behavior and cooperation with the chair in previous appointments. Children who were previously uncooperative (e.g., needing active or passive restraints) were often not recommended by the dentist to participate in the study. This might have been a source of selection bias as not all patients in the clinic would be eligible to participate in the study.

We likewise acknowledge that different dental procedures can cause different levels of DFA, for example, extractions involving local anesthesia injections might be more anxiety-inducing than a dental exam. No criteria were added regarding the type of dental treatment for this study because the study population is composed mostly of children with SHCN. For the general pediatric dental patients, a teeth cleaning procedure is usually deemed as an “easy” procedure, but it may be daunting for a child with SHCN. The effects of VR on these different procedures should be evaluated in future studies with a larger sample size.

## Conclusion

The results of the pilot study allow insight into the feasibility and acceptability of the VR intervention as well as the satisfaction of parents and health care professionals at a hospital dental clinic with a major population of children with SHCN. VR as a distraction method for children with SHCN was feasible and acceptable, and both parents and health care professionals’ satisfaction was high. Results can be used to guide future clinical trials on the use of VR in pediatric dentistry. By exploring new avenues of behavior and anxiety management tools for pediatric dentistry, this study and future studies will aim to provide improved patient-centered care.

## Data Availability

The authors confirm that the data supporting the findings of this study will be available through the repository Clinical Trials.

## Abbreviations Used

ASD: Autism spectrum disorder
ANCOVA: analysis of covariance
DFA: dental fear and anxiety
HCP: health care provider
REDCap: research electronic data capture
SHCN: special health care needs
VABRS: Venham Anxiety and Behavioral Rating Scales
VAS: visual analog scale
VR: virtual reality
